# Editorial: Computational Approaches for Human-Human and Human-Robot Social Interactions

**DOI:** 10.3389/frobt.2020.00055

**Published:** 2020-04-30

**Authors:** Cigdem Beyan, Vittorio Murino, Gentiane Venture, Agnieszka Wykowska

**Affiliations:** ^1^Pattern Analysis and Computer Vision, Istituto Italiano di Tecnologia, Genoa, Italy; ^2^Department of Computer Science, University of Verona, Verona, Italy; ^3^Huawei Technologies Ltd., Ireland Research Center, Dublin, Ireland; ^4^GV Lab., Department of Mechanical Systems Engineering, Tokyo University of Agriculture and Technology, Tokyo, Japan; ^5^Social Cognition in Human-Robot Interaction, Istituto Italiano di Tecnologia, Genoa, Italy

**Keywords:** human-human interaction, human robot interaction (HRI), machine learning, computational approaches, social interactions

## 1. Introduction

Non-verbal behaviors such as gaze, facial expressions, gestures, and vocal behavior carry significant information regarding human personality, emotions, engagement, intentions, action goals, and focus of attention. A large part of human communication takes place non-verbally (and often implicitly) during an explicit exchange of thoughts, attitudes, concerns, and feelings. Analyzing the basic principles of human communication through non-verbal signals is a long-standing research focus in cognitive and social psychology. However, the automatic realization of such analyses, especially by using machine learning (ML), or, in general, computational techniques, is a relatively unexplored avenue, although these techniques can be very efficient and effective.

Automatized detection and analysis of non-verbal social signals can be of particular relevance not only to human-human interaction (HHI) but also in human-robot interaction (HRI). Over the last decade, much research effort has been dedicated to improving robots' capabilities regarding perceiving, interacting, and cooperating with humans. Indeed, social HRI requires augmentation of robots' standard functionality with the ability to recognize and interpret human social signals in order to be able to engage naturally and intuitively with a human. Simultaneously, research efforts are being directed toward examining the human side of HRI, namely, the human mechanisms of social cognition in interactions with artificial agents (embodied robots specifically). This is crucial in order to understand how the human brain processes social signals coming from non-human agents and whether such agents can evoke mechanisms of social cognition in humans. ML techniques have also proved to be useful in this case to explore the patterns of neural and behavioral activity of the human counterparts.

This Research Topic is dedicated to exploring computational techniques for the analysis of non-verbal social signals in HHI as well as HRI. Specifically, we focus on ML methodologies, as well as other computational approaches for understanding non-verbal behavior and analyzing multi-modal data. It brings together ten selected papers that reflect some of the current computational approaches applied to HHI and HRI.

Bartlett et al. focus on movement analysis based on internal state identification. Video clips of social interactions, either the original scene or in the form of 2D body pose data, were shown to participants whose internal state perception was later assessed. These data were analyzed to determine whether the full scene clips were more informative than the 2D body pose. The results showed that participants were able to identify interaction imbalance, valence, and engagement independent of the types of videos. ML achieved similar performances as well, which can be interpreted as indicating that it can successfully decode and classify internal states using low-dimensional data.

Kory-Westlund and Breazeal investigate whether a social robot can increase children's rapport, positive emotion, acceptance, engagement, closeness, and learning. The robot entrained its speech and behavior to individual children and provided an appropriate backstory about its abilities. The data analysis performed showed that the robot's entrainment led children to show more positive emotions; it affected children's emulation of the robot's words in their own stories. Additionally, children who heard the robot's backstory were more accepting of it, find it more human-like, and agreed more to its requests.

Bloch et al. study the relevance of interpersonal synchrony (IS) for Autism Spectrum Disorder (ASD). IS is related to empathy and rapport and thus enables successful HHI, while individuals with ASD have difficulties with IS. The authors present a comprehensive review of IS in ASD and then propose a theoretical concept based on temporal processing of sensory input of interactions. Georgescu et al. present an ML method to study IS difficulties in ASD. IS between the head and upper body was quantified using Motion Energy Analysis, the results of which were used to train a Support Vector Machine to classify individuals with ASD and typically developed individuals.

Biancardi et al. propose a computational model that allows changes in the impression of warmth and competences of an embodied conversational agent that can interact with a human. The impressions of warmth and competence are changed in real-time to adapt to the human in order to maximize engagement. The system is tested as a museum guide, and it is shown that the hypothesis of warmth primacy may be valid.

Niewiadomski et al. focus on the analysis of social activities related to food and eating, as well as computational and technological approaches addressing such activities. The paper describes the approach of treating food-related activities as a social phenomenon that requires psychological and sociological analyses. It also presents problems that need to be tackled from the computational perspective, such as detection and recognition of food-related or eating activities.

Amiriparian et al. address interpersonal synchronization of acoustic signals during speech communication. They present an auto-encoder-based method trained on a large set of dyads across six different cultures. The results show that in all six cultures, partners tended to synchronize their speech, but inter-cultural differences were also observed.

Lammers et al. present a dataset of everyday actions expressing various emotions. This dataset was created based on motion capture data collected from human volunteers and then rendered into video files with a standardized, unified virtual character performing the actions. The stimulus material was then homogenized in terms of low-level physical features and tested for sufficiently high recognition rates.

Iwasaki et al. conducted an in-the-wild experiment, where a Pepper robot was in the role of a salesperson. The robot responded to various social situations and tried to attract customers' attention. Many customers ignored the robot's presence. However, if it managed to create a first impression of being capable of recognizing and appropriately responding to human behavior, it had higher chances of engaging customers. In a lab-environment experiment, the robot's “looking back behavior” was manipulated such that participants subjectively felt that they were being observed. The paper points out that for attracting the attention of users and maintaining their engagement, it is important to create an impression that a robot is aware of and reactive to the situational context, environment, and current state of the interaction.

Dinh et al. describe a framework for legible and safe robot behavior for HRI based on reinforcement learning. In a collaborative scenario, where both human and robot need to reach the same objects, the robot learns how to be legible to the human and how to avoid dynamic obstacles, thereby improving the safety of the human. This was tested in a virtual reality setup and in a physical HRI with a KUKA robot arm. The results showed that over the course of the experiment, participants efficiently learned how to predict robot movements and rated the robot's legibility increasingly higher. That improvement was better compared to a non-adaptive condition. The important advantage of this approach is that it is generalizable to other tasks.

## Author Contributions

All co-authors drafted, revised the manuscript for important intellectual content, and approved the final version to be published.

## Conflict of Interest

VM is employed by the company Huawei Technologies. The remaining authors declare that the research was conducted in the absence of any commercial or financial relationships that could be construed as a potential conflict of interest.

